# Burden of prolonged treatment delay among patients with common cancers in the Philippines

**DOI:** 10.1007/s10552-025-01969-6

**Published:** 2025-02-24

**Authors:** Jansen M. Cambia, Arnat Wannasri, Edmund Cedric A. Orlina, Gehan Alyanna C. Calvez, Wilma M. Grafilo, Jason J. Liu

**Affiliations:** 1 International Health Program, National Yang Ming Chiao Tung University, Taipei, Taiwan; 2 Department of Health- Rizal Cancer Registry, Rizal Medical Center, Metro Manila, Philippines; 3 Department of Medical Services, Ministry of Public Health, Nopparat Rajathanee Hospital, Bangkok, Thailand; 4 Institute of Public Health, National Yang Ming Chiao Tung University, No.155, Sec. 2, Linong St, Beitou District, Taipei City, 112 Taiwan

**Keywords:** Treatment delay, Cancer, Cancer registry, Population-based study, Epidemiology

## Abstract

**Purpose:**

Prolonged treatment delay often leads to adverse cancer prognosis. However, the demographic and clinical predictors of higher treatment delay burden in the Philippines have not been thoroughly evaluated.

**Methods:**

We conducted a population-based retrospective cohort study on patients diagnosed with common cancers who received cancer treatment, to quantify the burden of prolonged treatment delay in the Philippines among this population. We analyzed 20,654 patients with common cancers from the Department of Health-Rizal Cancer Registry. The Poisson regression model with robust variance was used to identify demographic and clinical predictors of prolonged treatment delay. In addition, we examined the associations among those receiving different initial treatment types, including surgery, radiotherapy, and chemotherapy.

**Results:**

We found 35.1 % of the studied cancer patients experienced initial treatment delay of more than 30 days, as well as 25.2 % and 20.0 % experiencing treatment delays exceeding 60 and 90 days, respectively. We found higher risk of prolonged treatment delay of more than 90 days in those with 0–19 years of age at diagnosis, male gender, cancer treatment at non-private hospitals, diagnoses during the 1990s, more advanced cancer stages, and non-surgical initial treatments. For patients with surgery as the initial treatment, younger age at cancer diagnosis was not significantly associated with increased burden of prolonged treatment delay, unlike for those initially treated with radiotherapy or chemotherapy.

**Conclusion:**

By identifying the characteristics of treated cancer patients with higher risk of protracted treatment delay, our findings will inform the national cancer control program to especially target those patients for treatment delay reduction.

**Supplementary Information:**

The online version contains supplementary material available at 10.1007/s10552-025-01969-6.

## Introduction

In February 2019, the Philippines signed into law the National Integrated Cancer Control Act (NICCA). One of its aims was to enhance patient navigation in cancer care, ensuring that patients receive timely cancer management from their initial interaction with healthcare workers until the completion of their treatment [1]. It has been recommended that cancer patients initiate treatment within 30 days and not exceed 90 days from diagnosis [2], but around 33 % of cancer patients experienced treatment delay exceeding four weeks [3]. Prolonged delay of receiving initial surgery or radiotherapy/systemic therapy for more than 30 days among cancer patients reduced survival by 6 % to 8 % and 9 % to 13 %, respectively [4]. A previous study in the Philippines revealed that delays in seeking inpatient and outpatient care were attributed to non-urgent healthcare needs, age at diagnosis, educational background, job status, income, and inadequate healthcare utilization [5]. Although there have been studies on the predictors of treatment delay in the general population and for patients with specific cancers such as cervical, breast, and colorectal cancer [5–9], limited research has quantified the risk of treatment delay in a broader cancer patient population. By identifying the demographic and clinical characteristics of patients with common cancers prone to prolonged treatment delays, our findings may encourage efforts to mitigate the high prevalence of treatment delay among these patients.

Using the first population-based cancer registry in the Philippines, we conducted a study to understand the demographic and clinical predictors of prolonged treatment delays of > 30 days, > 60 days, and > 90 days among patients with common cancers who received cancer treatment. In addition, we examined the predictors of prolonged treatment delay stratified by initial treatment types.

## Methods

### Data sources

We obtained our data from the Department of Health—Rizal Cancer Registry (DOH-RCR) in Pasig City, the Philippines. The DOH-RCR was established in 1974 and was the first population-based cancer registry in the country. Covering 1,343 km^2^, it spans 26 cities and municipalities across 14 regions in Rizal Province and 12 districts in the National Capital Region. By 2012, this area covered roughly 7 million people, or about 8.0 % of the nation's total population [10]. Since 1980, DOH-RCR has operated active surveillance by gathering patients’ demographic and cancer-related data at 131 hospitals as well as 26 Directory of Civil Registrars (https://psa.gov.ph/lcr-directory) [11].

The International Agency for Research on Cancer (IARC) recognized DOH-RCR met several criteria for a high-quality registry, which included the following criteria: (a) for data comparability, the cancer registry has followed the International Classification of Diseases for Oncology, Third Edition (ICD-O-3), ensuring consistent classification and coding of new cases; (b) for data validity, CanReg4 Software has been used to perform consistency checks during data entry; and (c) for data completeness, recent data quality indices reported by the registry have shown a substantial proportion of microscopically verified cancer cases (68 % in men and 79 % in women) between 2003 and 2007, surpassing cases reported through death certificates. These percentages rose to 84 % for men and 83 % for women from 2008 to 2012 [12].

Patient identities remained anonymous to the researcher and were replaced with identification numbers. In addition, patient-level data were not shared publicly but could be accessed upon request to the DOH-RCR, subject to Institutional Review Board approval. This study followed Helsinki Declaration principles and was authorized by the Rizal Medical Center Institutional Review Board (IRB number: 2022-Z-#023-RP-1. II) and National Yang Ming Chiao Tung University Institutional Review Board (IRB number: YM111009E).

### Study design and population

This was a population-based retrospective cohort study of 20,654 cancer patients. The study utilized the DOH-RCR dataset spanning 23 years of follow-up from 1990 to 2012, the most recent year of data available in the registry for analysis. The study population consisted of individuals who were diagnosed with and received initial treatment for the ten most common cancer types in the Philippines, which was identified by the Global Cancer Observatory in 2020 [13]. These cancers defined using ICD-O-3 included cancers of the breast (C50) in females; trachea, bronchus, and lung (C33-34); colon and rectum (C18-21); liver and intrahepatic bile ducts (C22); cervix uteri (C53); prostate (C61); thyroid (C73); stomach (C16); and ovary (C56) [14]; as well as leukemia (C421). We excluded those who did not undergo cancer treatment or had missing information on initial treatment. Additionally, we focused exclusively on invasive cancers, excluding in-situ carcinoma.

### Statistical analysis

We used Poisson regression with robust variance to calculate the risk ratio (RR) and 95 % confidence interval (CI), for identifying factors that significantly predicted prolonged treatment delay of > 30 days, > 60 days, and > 90 days. Treatment delay was defined as the time interval from the date of cancer diagnosis to the date of the patient’s first cancer treatment. The demographic predictors we examined included: age at diagnosis (0–19, 20–39, 40–64, 65 + years); sex (male, female); residence location (city, municipality); hospital type for cancer treatment (private hospitals, not hospitalized, specialty hospitals, non-government organizations, local government hospitals, national government hospitals); and year at diagnosis (1990–1999, 2000–2012). The clinical predictors we examined included: cancer stages (stage I, stage II, stage III, stage IV), initial treatment type (surgery, radiotherapy, chemotherapy), and cancer type (thyroid, breast, lung, colorectal, liver, cervical, prostate, leukemia, stomach, ovary). For the analysis involving cancer type, we chose thyroid cancer as the reference group because it had the best survival prognosis compared with the other common cancers. We also stratified our findings by first treatment type (surgery, radiotherapy, or chemotherapy), year at diagnosis (1990–1999, 2000–2012), and hospital type for cancer treatment (private hospitals, non-government organizations, local government hospitals, national government hospitals).

We conducted a sensitivity analysis using multiple imputation by chained equations to impute variables with missing data, including treatment delay, residence location, hospital type, cancer stage, and initial treatment type [15, 16]. We generated 20 imputed datasets for each missing variable, as more than 10 % of the participants had missing information [16, 17].

All analyses were conducted using STATA 15.0 (StataCorp, TX, USA). Two-tailed *p* value < 0.05 indicated statistical significance.

## Results

The characteristics of the study participants are shown in Table 1. Among the 20,654 study participants, most were 40–64 years of age (63.1 %), females (81.2 %), living in cities (84.2 %), admitted to private hospitals (58.0 %), diagnosed with stage III cancer (32.2 %), and treated with surgery as the first treatment (70.2 %). In addition, the most common cancers were breast, cervical, thyroid, lung, ovarian, and prostate cancers.

**Table 1:** Descriptive statistics of the predictive factors of treatment delay between 1990 and 2012

Variables	No. of Samples (*n* = 20,654)	Percentage
*Age at diagnosis*		
0–19 years	366	1.77
20–39 years	3,233	15.65
40–64 years	13,025	63.06
65 + years	4,030	19.51
*Sex*		
Female	16,779	81.23
Male	3,875	18.77
*Residence location*		
City	17,346	84.19
Municipality	3,259	15.81
*Hospital types*		
Private	11,977	57.99
Not Hospitalized	157	0.76
Specialty	646	3.13
Non-Governmental Organization	3,521	17.05
Local Government	901	4.36
National Government	3,452	16.71
*Year at diagnosis*		
1990–1999	4,156	20.12
2000–2012	16,498	79.88
*Cancer stages*		
Stage I	4,596	29.88
Stage II	2,352	15.29
Stage III	4,944	32.15
Stage IV	3,488	22.68
*Initial treatment*		
Surgery	14,120	70.21
Radiotherapy	3,961	19.70
Chemotherapy	2,030	10.09
*Cancer types*		
Thyroid	2,337	11.31
Breast	9,932	48.10
Lung	1,549	7.50
Colorectal	538	2.60
Liver	195	0.94
Cervical	2,394	11.59
Prostate	1,287	6.23
Leukemia	450	2.18
Stomach	448	2.17
Ovarian	1,524	7.38

In Table 2, we found 35.1 % of patients experienced treatment delay of more than 30 days, followed by 25.2 % and 20.0 %, respectively, experiencing delay of more than 60 and 90 days. Prolonged treatment delay of more than 90 days was significantly associated with 0–19 years of age at diagnosis, male gender, treatment at non-private hospitals, diagnoses during the 1990s, higher cancer stages, and receiving non-surgical initial treatments. Those with leukemia, lung cancer, liver cancer, and cervical cancer had the highest risk of prolonged treatment delay. The factors predicting treatment delay were mostly consistent for treatment delays of more than 30, 60, and 90 days. Supplementary Table 1 presents the results from analyzing the imputed overall population, which showed mostly consistent significant predictors of prolonged treatment delay.

**Table 2:** Univariate analysis on the predictors of treatment delay among patients with common cancers

Variables	> 30 Days of Delay			> 60 Days of Delay			> 90 Days of Delay		
	*Delay* (%) *n*= 7,348 (35.1 %)	RR	95 *%* CI	*Delay* (%) *n* = 5,287 (25.2 %)	RR	95 % CI	*Delay* (%) *n* = 3,980 (20.0 %)	RR	95 *%* CI
*Age at diagnosis*									
0–19 years	47.0	1		39.6	1		35.5	1	
20–39 years	33.0	0.70*	0.62 – 0.79	24.5	0.62*	0.54 – 0.71	19.1	0.54*	0.46 – 0.63
40–64 years	36.0	0.77*	0.69 – 0.86	25.7	0.65*	0.57 – 0.74	20.3	0.57*	0.49 – 0.66
65 + years	35.2	0.75*	0.67 – 0.84	24.9	0.63*	0.55 – 0.72	19.8	0.56*	0.48 – 0.65
*Sex*									
Female	34.4	1		24.4	1		19.1	1	
Male	40.6	1.18*	1.13 – 1.23	30.8	1.26*	1.20 – 1.33	25.7	1.35*	1.27 – 1.44
*Residence location*									
City	35.4	1		25.6	1		20.4	1	
Municipality	36.7	1.04*	1.00 – 1.09	25.8	1.01	0.95 – 1.08	19.7	0.96	0.89 – 1.04
*Hospital types*									
Private	30.0	1		21.6	1		17.6	1	
Not Hospitalized	46.5	1.55*	1.31 – 1.84	30.0	1.39*	1.09 – 1.77	23.6	1.34*	1.01 – 1.79
Specialty	36.0	1.20*	1.08 – 1.34	26.9	1.25*	1.09 – 1.42	21.8	1.24*	1.06 – 1.44
Non-Governmental Organization	42.2	1.41*	1.34 – 1.48	31.5	1.46*	1.38 – 1.55	25.3	1.44*	1.35 – 1.55
Local Government	34.6	1.15*	1.05 – 1.27	23.1	1.07	0.94 – 1.21	17.1	0.97	0.84 – 1.13
National Government	47.7	1.59*	1.52 – 1.66	33.7	1.56*	1.47 – 1.66	25.0	1.42*	1.33 – 1.52
*Year at diagnosis*									
1990–1999	34.8	1		26.1	1		21.5	1	
2000–2012	35.7	1.02	0.97 – 1.07	25.4	0.97	0.91 – 1.02	19.9	0.92*	0.86 – 0.98
*Cancer stages*									
Stage I	26.3	1		17.5	1		13.0	1	
Stage II	32.4	1.23*	1.14 – 1.33	22.6	1.29*	1.17 – 1.43	17.6	1.35*	1.21 – 1.51
Stage III	31.5	1.20*	1.12 – 1.28	21.5	1.23*	1.13 – 1.33	16.6	1.27*	1.16 – 1.41
Stage IV	46.3	1.76*	1.66 – 1.87	35.7	2.04*	1.89 – 2.20	29.6	2.27*	2.08 – 2.49
*Initial treatment*									
Surgery	23.9	1		16.3	1		12.6	1	
Radiotherapy	60.7	2.54*	2.45 – 2.64	44.8	2.74*	2.60 – 2.88	36.0	2.85*	2.68 – 3.02
Chemotherapy	62.4	2.61*	2.50 – 2.73	46.9	2.87*	2.70 – 3.04	38.1	3.02*	2.81 – 3.24
*Cancer types*									
Thyroid	20.9	1		15.2	1		12.0	1	
Breast	33.9	1.62*	1.49 – 1.76	24.5	1.61*	1.44 – 1.77	19.4	1.62*	1.43 – 1.81
Lung	50.9	2.43*	2.22 – 2.67	38.4	2.52*	2.25 – 2.83	31.8	2.65*	2.32 – 3.02
Colorectal	24.5	1.17*	1.00 – 1.39	17.1	1.12	0.91 – 1.38	13.9	1.16	0.91 – 1.47
Liver	44.1	2.11*	1.77 – 2.51	33.9	2.22*	1.79 – 2.76	28.7	2.39*	1.87 – 3.06
Cervical	53.9	2.57*	2.34 – 2.79	35.1	2.31*	2.05 – 2.56	25.4	2.11*	1.84 – 2.38
Prostate	37.9	1.81*	1.62 – 2.00	28.4	1.87*	1.63 – 2.11	22.8	1.89*	1.62 – 2.19
Leukemia	60.4	2.89*	2.59 – 3.22	49.1	3.22*	2.82 – 3.69	43.1	3.59*	3.08 – 4.18
Stomach	29.9	1.43*	1.22 – 1.68	20.1	1.32*	1.07 – 1.62	14.7	1.23	0.96 – 1.57
Ovarian	20.1	0.96	0.84 – 1.08	15.1	0.99	0.85 – 1.15	12.9	1.08	0.90 – 1.27

*p value < 0.05

We further identified the factors predicting prolonged treatment delay of more than 90 days among those receiving initial surgery, radiotherapy, or chemotherapy treatment in Table 3. The percentage of patients experiencing treatment delay for initial chemotherapy treatment was 38.1 %, followed by radiotherapy and surgery at 36.0 % and 12.6 %, respectively. For patients who underwent initial surgery, age at cancer diagnosis was not significantly associated with prolonged treatment delay, unlike for those receiving radiotherapy or chemotherapy as initial treatment. Male patients who underwent initial surgery had significantly higher risk of prolonged treatment delay than females. Over 40 % of leukemia patients had treatment delay of more than 90 days, regardless of initial treatment type. For the other common cancers, the percentage of treatment delay of more than 90 days was below 20 % for those with initial surgery, whereas the percentage was generally above 30 % for those with radiotherapy or chemotherapy as initial treatment.

**Table 3:** Univariate analysis on the predictors of treatment delay of more than 90 days among patients with common cancers, stratified by the initial treatment types

Variables	Surgery			Radiotherapy			Chemotherapy		
	*Delay* (%) *n* = 1,781 (12.6 %)	RR	95 *%* CI	*Delay* (*%*) *n* = 1,425 (36.0 %)	RR	95 *%* CI	*Delay* (*%*) *n* = 774 (38.1 %)	**RR**	95 *%* CI
*Age at diagnosis*									
0–19 years	12.2	1		53.1	1		47.3	1	
20–39 years	12.3	1.00	0.63 – 1.60	41.8	0.79	0.56 – 1.12	42.0	0.89	0.72 – 1.09
40–64 years	12.7	1.03	0.88 – 1.64	36.3	0.68*	0.49 – 0.95	36.8	0.78*	0.66 – 0.91
65 + years	12.8	1.04	0.66 – 1.67	31.8	0.60*	0.43 – 0.84	34.9	0.74*	0.60 – 0.90
Sex									
Female	12.4	1		35.3	1		38.1	1	
Male	14.0	1.13*	1.00 – 1.27	37.6	1.07	0.98 – 1.17	38.0	1.00	0.99 – 1.13
*Residence location*									
City	12.8	1		35.9	1		37.8	1	
Municipality	11.8	0.92	0.81 – 1.03	36.1	1.00	0.90 – 1.14	39.5	1.04	0.91 – 1.21
*Hospital types*									
Private	10.7	1		32.9	1		32.9	1	
Not Hospitalized	14.0	1.31	0.79 – 2.18	40.0	1.21	0.83 – 1.79	28.6	0.87	0.44 – 1.73
Specialty	9.7	0.91	0.66 – 1.22	36.4	1.11	0.87 – 1.42	44.4	1.35*	1.07 – 1.72
Non-Governmental Organization	17.4	1.63*	1.47 – 1.82	39.5	1.20*	1.07 – 1.34	50.5	1.54*	1.34 – 1.77
Local Government	12.4	1.16	0.95 – 1.41	41.3	1.25	0.93 – 1.69	38.0	1.16	0.85 – 1.58
National Government	15.3	1.44*	1.28 – 1.62	39.8	1.21*	1.09 – 1.33	44.9	1.37*	1.19 -1.58
*Year at diagnosis*									
1990–1999	14.9	1		34.1	1		40.5	1	
2000–2012	12.0	0.80*	0.72 – 0.89	36.5	1.06	0.96 – 1.18	37.6	0.92	0.80 – 1.06
*Cancer stages*									
Stage I	10.0	1		33.7	1		28.0	1	
Stage II	11.3	1.13	0.96 – 1.33	31.5	0.94	0.78 – 1.13	38.5	1.38	0.99 – 1.92
Stage III	11.7	1.17*	1.03 – 1.32	43.1	1.28*	1.08 – 1.52	36.0	1.28	0.95 – 1.74
Stage IV	17.8	1.78*	1.54 – 2.05	37.2	1.10	0.95 – 1.29	40.4	1.44*	1.10 – 1.89
*Cancer types*									
Thyroid	9.7	1		45.9	1		38.9	1	
Breast	13.4	1.38*	1.20 – 1.59	54.6	1.19	0.96 – 1.44	39.5	1.02	0.56 – 1.81
Lung	19.0	1.97*	1.42 – 2.72	32.5	0.71*	0.57 – 0.88	34.6	0.89	0.49 – 1.61
Colorectal	8.4	0.87	0.63 – 1.21	55.3	1.20	0.85 – 1.70	36.6	0.94	0.46 – 1.91
Liver	17.1	1.76*	1.08 – 2.89	33.3	0.73	0.47 – 1.12	34.0	0.87	0.44 – 1.74
Cervical	16.2	1.67*	1.33 – 2.07	27.3	0.60*	0.48 – 0.73	31.8	0.82	0.44 – 1.53
Prostate	15.7	1.62*	1.32 – 1.97	37.5	0.82	0.65 – 1.03	50.0	1.29	0.66 – 2.51
Leukemia	41.7	4.31*	2.87 – 6.47	42.4	0.92	0.68 – 1.25	43.1	1.10	0.61 – 2.01
Stomach	11.5	1.19	0.87 – 1.62	26.1	0.57	0.28 – 1.16	33.3	0.86	0.42 – 1.75
Ovarian	9.1	0.94	0.76 – 1.16	53.7	1.17	0.85 – 1.60	34.0	0.87	0.46 – 1.66

*p value < 0.05

In Supplementary Table 2, we identified factors predicting prolonged treatment delays of more than 90 days, stratified by year at diagnosis. Patients diagnosed in either the 1990s or 2000s who were pediatric, males, treated in non-private hospitals, had metastatic cancer, or underwent non-surgical treatment were at higher risk for prolonged treatment delays.

We also identified the predictors of prolonged treatment delays of more than 90 days stratified by residence location. The predictors for patients residing in the city and municipality were largely consistent, except the risk of treatment delay significantly reduced in 2000–2012 compared with 1990–1999 in city residents but not municipality residents. Also, a larger proportion of male patients had treatment delay in the city compared with in the municipality (Supplementary Table 3).

The analysis results for predictors of prolonged treatment delays of more than 90 days stratified by hospital type in Supplementary Table 4 showed that the significant predictors were consistent across all hospital types, including pediatric age at diagnosis, male sex, advanced cancer stages, or non-surgical initial treatment.

## Discussion

To our knowledge, this is the first study to assess the burden of treatment delay among patients with common cancers who received treatment in the Philippines. We found 35.1 % of the patients experienced initial treatment delay of more than 30 days, as well as 25.2 % and 20.0 % experiencing treatment delays exceeding 60 and 90 days, respectively. We also found significantly higher risk of prolonged treatment delay among patients with 0–19 years of age at diagnosis, male sex, diagnoses during the 1990s, cancer treatment at non-private hospitals, advanced cancer stages, and non-surgical initial treatments.

In the Philippines, cancer patients face challenges accessing healthcare. Geographic barriers and unaffordable conventional therapies are obstacles of timely treatment in the country. The Philippines is deeply rooted in traditional theories of illness causation, and traditional medicine remains popular among cancer patients. Therefore, patients often explore scientifically unproven treatment options, such as herbal concoctions, prayers, and rituals performed by local shaman called “Babaylan.” When the patients’ conditions worsen after trying these alternative treatments, they turn to hospitals and seek conventional medical help, causing prolonged treatment delays, higher healthcare costs, and premature deaths [18, 19].

Previous studies assessing treatment delays in the general population and specific cancer types, such as cervical, breast, and colorectal cancer have identified several common factors that influenced treatment delay. These factors included high healthcare costs, transportation issues, marital status, age, sex, cancer stage, patients’ knowledge and education, household income, and lack of health insurance [5–9]. Younger patients were more likely to delay medical care compared with older adults and the elderly [6]. Males experienced more treatment delay than women did [9]. Also, there was a significant association between late cancer stage and prolonged treatment delay [7]. A study of Bangladeshi breast cancer patients found those who used alternative medicine were four times more likely to experience delays in seeking conventional medical assistance [18].

In this study, pediatric patients had higher risk of treatment delay than older patients. Treatment delay among pediatric cancer patients is often attributed to parental care, because pediatric patients rely on their parents for healthcare decision making [20]. In high-income countries, over 80 % of children diagnosed with cancer survive their illness, as cure-directed therapy is routinely provided at the timely diagnosis for most patients and their families. However, the global burden of pediatric cancer is disproportionately distributed, with approximately 90 % of cases occurring in low- and middle-income countries (LMIC), including the Philippines [21]. Numerous policy gaps in LMICs were identified that exacerbated treatment delays among children diagnosed with cancer [22]. These gaps included the lack of childhood cancer care resources and access to healthcare [22]. Parents with lower socioeconomic and educational status were at higher risk of delaying treatment for their children [20]. The parental decision to use traditional medicine may also prolong treatment delay for pediatric cancer patients [23].

We found extended treatment delays were more common among males. A previous study had consistent results on the male susceptibility for cancer treatment delay [24]. A Canadian study revealed that women were more inclined to proactively seek help, resulting in more frequent engagement with the healthcare system [25]. Prior research suggested that men who discussed their cancer diagnosis with partners and friends more actively sought curative treatments [26, 27]. However, relying on family and friends can lead to difficulties in making treatment decisions because of conflicting opinions and preferences [26]. Therefore, it is important to also consult healthcare professionals to make treatment decisions.

Our study found prolonged treatment delay among late-stage cancer patients. A previous study revealed that advanced cancer stage and worse cancer prognosis can influence temporary treatment refusal and treatment delay [19]. This might be due to the patients' frail physical state and perceived difficulty in enduring physical discomfort during treatment. However, as the disease progresses, some patients may opt for treatments to alleviate their pain and symptoms [19, 28]. Furthermore, patients with more advanced cancer stages were more likely to pursue traditional medicine, potentially leading to treatment delay [18].

Our study showed that the percentage of cancer treatment delay of more than 90 days in the Philippines reduced in 2000–2012 compared with in 1990–1999. Several policies implemented during these years may have influenced the improvement in cancer treatment care [1, 29–31]. The early establishment of the Philippine Cancer Control Program (PCCP) in 1988 provided guidelines for cancer control, specifying program policies, components, and implementation timelines. The PCCP advocated prompt referrals for detected cases and timely treatment [29]. In 1992, the PCCP gradually expanded its coverage to include all regions of the country through the designation of Regional and Municipal Cancer Control Coordinators, which eventually led to the establishment of the Community-Based Cancer Care/Cancer Network in 1998 [29]. During this time, the implementation of the Specific Cancer-Site Cancer Control Program was also initiated [30]. The Philippine government developed additional national programs for cancer control, such as the Breast Cancer Control Program and the Lung Cancer Control Program, along with targeted programs for other common cancers, including cervical, liver, colorectal, and prostate cancers [29, 30]. The strengthening of programs close to the 2000s may have contributed to improvements in cancer care and timely treatment in the subsequent years.

To continually improve cancer care in the Philippines, the government transformed the PCCP into the National Cancer Prevention and Control Action Plan of 2015–2020 [31]. This also led to the development of NICCA, which includes wide-ranging provisions covering the establishment of national and regional cancer centers, educational initiatives for both healthcare professionals and the general public, the creation of a national cancer registry, and financial support for cancer research and cancer patients [1]. The 2024–2028 National Integrated Cancer Control Program Strategic Framework was also launched. It continues the vision and mission of NICCA, while underscoring the significance of setting priorities and strengthening capacities to achieve cancer control. A key aspect of the framework is the emphasis on strengthening universal health coverage and bolstering primary healthcare systems to ensure equitable access to affordable and high-quality care. This is particularly crucial for improving treatment delivery for common cancers [32]. Overall, these initiatives aimed to improve cancer prevention, cancer detection, and treatment capabilities to benefit the population. Furthermore, the programs sought to develop systems for monitoring and evaluating the effectiveness of these interventions.

We also found greater treatment delay among patients who visited non-private hospitals compared with private ones. In 2009, private hospitals in the Philippines outnumbered public ones by about 60 %, but only around 48 % of those seeking inpatient care utilized private hospitals [34]. A study found higher patient volume led to longer waiting lists and treatment delays for cancer patients [35]. The healthcare facilities in the Philippines are relatively adequate in metropolitan areas, where most of the private hospitals are located, whereas non-private hospitals lack healthcare resources and infrastructures. Nonetheless, many Filipinos heavily rely on public and government-owned hospitals as they provide lower cost of healthcare compared with private hospitals [36].

Accessing all types of hospitals in the Philippines can be challenging due to transportation issues, particularly when most hospitals are located far from residential areas. The archipelagic structure of the Philippines, comprising over 7,500 islands, presents challenges in establishing comprehensive transportation networks for reaching major hospitals providing cancer care and treatment [1, 37]. To strengthen the inter-island linkages, the government implemented the Roll-on/Roll-off (Ro-Ro) policy in 2003 through the establishment of the Ro-Ro ferry Terminal System. Furthermore, the Philippine Development Plan (PDP) 2011–2016 focused on infrastructure development, including the transportation sector [37]. However, the implementation of the PDP encountered challenges, including the lack of integration between national and local government plans and programs. This disconnect has resulted in gaps in the transport network, contributing to low capacity and quality of infrastructure facilities. These challenges can hinder easy transportation access for some patients, potentially impeding their ability to access timely and convenient health care [37]. Even without these challenges, patients living in more remote regions, especially those in the lowest income category, rely on nearby non-governmental organization and local government hospitals for initial cancer treatment, since transportation is a major problem when accessing hospitals with complete facilities [38]. However, for therapies that require more advanced procedures and specialized oncologists, patients may need to follow a referral pathway to national government facilities, which can lead to prolonged treatment initiation [38].

Prolonged treatment delays can also result from the lack of staff and hospital facilities for cancer treatments [39]. A global study recommended that for a LMIC like the Philippines, the optimal lower bound for the medical oncologist workforce should be 1.25 per 100,000 people [40], whereas the current medical oncologist workforce in the country stands at 0.32 per 100,000 people [39]. Also, the global benchmark for radiation oncologists is 0.28 per 100,000 people [40], whereas there are 0.09 per 100,000 people in the Philippines [39]. Limited radiation therapy facilities hinder radiation oncologists to perform their tasks promptly, prolonging cancer treatment delay. Out of 50 radiation therapy facilities in the Philippines, 19 are located in Metro Manila, while the remaining 31 are situated in the provinces. Similarly, among 25 brachytherapy facilities, 10 are in Metro Manila, and 15 are distributed in provinces [39].

Our study has several strengths. First, there have been limited studies investigating treatment delays for common cancers such as leukemia and cancers of the liver, thyroid, stomach, prostate, and ovary. Our study includes these common cancers, allowing us to understand the burden of prolonged treatment delay in a broader population of cancer patients. Second, to our knowledge, previous studies have not compared the predictors of prolonged treatment delay across various initial treatment modalities, including surgery, radiotherapy, and chemotherapy. However, this study provides the risk of treatment delay by demographic and clinical factors for those with different initial treatment types. Lastly, the IARC rated DOH-RCR as a high-quality population-based cancer registry that met stringent standards of validity, accuracy, and completeness of coverage.

However, this study also has some limitations. We could only study treatment delay in patients who received treatment and had treatment information. Many elderly patients did not have treatment records, likely due to treatment refusal and that certain treatments were not recommended for them. Therefore, our findings may not be generalizable to all patients in the DOH-RCR. Another drawback of our study was the inability to distinguish between patient-level and system-level treatment delay lengths. We recommend that future studies utilizing primary data collection examine both patient decision-making processes and healthcare system delays. Furthermore, we were unable to assess other important predictors of treatment delay, such as patient’s lifestyle and behavioral factors, comorbidities, and socioeconomic status. Although our study is informative for understanding the risk and burden of cancer treatment delay in patients of various demographic and clinical characteristics, future studies are needed to understand the causal relationships between these factors and treatment delay.

## Conclusion

In this study, we quantified the risk of prolonged treatment delay among treated cancer patients in the Philippines by important demographic and clinical factors. Because treatment delay can adversely affect the prognosis of cancer patients, minimizing treatment delay can improve survival and quality of life. Our findings may improve the Philippines' cancer control program by encouraging efforts to reduce the burden of treatment delay among patients with common cancers. We recommend targeting cancer patients at higher risk of prolonged treatment delays, such as pediatric patients, those with more advanced cancer stages, those receiving non-surgical initial treatments, and those relying on non-private hospitals.

## Supplementary Information

Below is the link to the electronic supplementary material.

Supplementary file1 (DOCX 61 KB)

## Data Availability

The data for this study can be obtained from the Department of Health-Rizal Cancer Registry (DOH-RCR) by applying and adhering to the requirements of the Rizal Medical Center Institutional Review Board. The data supporting the findings of this study can be made available upon reasonable request from the corresponding author.

## Abbreviations

CI: Confidence intervals
DOH-RCR: Department of Health–Rizal Cancer Registry
IARC: International Agency for Research on Cancer
ICD-O-3: International Classification of Diseases for Oncology, Third Edition
LMIC: Low- and middle-income countries
NICCA: National Integrated Cancer Control Act
RR: Risk ratios
Ro-Ro: Roll-on/Roll-off
PCCP: Philippine Cancer Control Program
PDP: Philippine Development Plan
