# Supplementary material for: Burden of prolonged treatment delay among patients with common cancers in the Philippines
Source: Cancer Causes Control. 2025 Feb 24;36(7):663–72. doi: 10.1007/s10552-025-01969-6 (PMC12103467; doi:10.1007/s10552-025-01969-6)
Supplement: Supplementary file 1 — Supplementary file1 (DOCX 61 KB) [file 10552_2025_1969_MOESM1_ESM.docx]

**Supplementary Table 1. Univariate analysis on the predictors of treatment delay among patients with common cancers, after applying multiple imputation**

| Variables | >30 Days of Delay | | >60 Days of Delay | | >90 Days of Delay | |
| --- | --- | --- | --- | --- | --- | --- |
|  | RR | 95*%* CI | RR | 95*%* CI | RR | 95*%* CI |
| *Age at diagnosis* |  |  |  |  |  |  |
| 0-19 years | 1 |  | 1 |  | 1 |  |
| 20-39 years | 0.79* | 0.75 – 0.83 | 0.76* | 0.71 – 0.82 | 0.75* | 0.69 – 0.80 |
| 40-64 years | 0.80* | 0.76 – 0.84 | 0.76* | 0.72 – 0.81 | 0.75* | 0.70 – 0.80 |
| 65+ years | 0.81* | 0.77 – 0.86 | 0.77* | 0.72 – 0.82 | 0.76* | 0.70 – 0.82 |
| *Sex* |  |  |  |  |  |  |
| Female | 1 |  | 1 |  | 1 |  |
| Male | 1.08* | 1.05 – 1.11 | 1.09* | 1.05 – 1.13 | 1.11* | 1.06 – 1.16 |
| *Residence location* |  |  |  |  |  |  |
| City | 1 |  | 1 |  | 1 |  |
| Municipality | 1.04 | 0.99 – 1.09 | 1.01 | 0.95 – 1.07 | 0.95 | 0.88 – 1.03 |
| *Hospital types* |  |  |  |  |  |  |
| Private | 1 |  | 1 |  | 1 |  |
| Not Hospitalized | 1.62* | 1.40 – 1.87 | 1.52* | 1.23 – 1.87 | 1.46* | 1.13 – 1.88 |
| Specialty | 1.20* | 1.05 – 1.37 | 1.25* | 1.06 – 1.47 | 1.24* | 1.01 – 1.53 |
| Non-Governmental Organization | 1.39* | 1.33 – 1.46 | 1.46* | 1.38 – 1.55 | 1.45* | 1.36 – 1.55 |
| Local Government | 1.19* | 1.10 – 1.28 | 1.13* | 1.03 – 1.25 | 1.04 | 0.90 – 1.19 |
| National Government | 1.57* | 1.51 – 1.63 | 1.57* | 1.49 – 1.65 | 1.44* | 1.36 – 1.53 |
| *Year at diagnosis* |  |  |  |  |  |  |
| 1990 – 1999 | 1 |  | 1 |  | 1 |  |
| 2000 – 2012 | 1.04* | 1.01 – 1.06 | 1.02 | 0.99 – 1.06 | 1.02 | 0.98 – 1.06 |
| *Cancer stages* |  |  |  |  |  |  |
| Stage I | 1 |  | 1 |  | 1 |  |
| Stage II | 1.17* | 1.10 – 1.25 | 1.22* | 1.10 – 1.35 | 1.28* | 1.14 – 1.44 |
| Stage III | 1.21* | 1.13 – 1.30 | 1.25* | 1.14 – 1.37 | 1.30* | 1.17 – 1.46 |
| Stage IV | 1.77* | 1.63 – 1.92 | 1.98* | 1.77 – 2.20 | 2.16* | 1.90 – 2.47 |
| *Initial treatment* |  |  |  |  |  |  |
| Surgery | 1 |  | 1 |  | 1 |  |
| Radiotherapy | 2.46* | 2.37 – 2.56 | 2.62* | 2.52 – 2.79 | 2.75* | 2.57 – 2.93 |
| Chemotherapy | 2.51* | 2.40 – 2.63 | 2.76* | 2.59 – 2.93 | 2.90* | 2.70 – 3.11 |
| *Cancer types* |  |  |  |  |  |  |
| Thyroid | 1 |  | 1 |  | 1 |  |
| Breast | 1.19* | 1.12 – 1.25 | 1.18* | 1.10 – 1.26 | 1.18* | 1.10 – 1.27 |
| Lung | 1.36* | 1.27 – 1.46 | 1.37* | 1.27 – 1.48 | 1.40* | 1.28 – 1.52 |
| Colorectal | 1.04 | 0.93 – 1.16 | 1.01 | 0.88 – 1.17 | 1.04 | 0.89 – 1.21 |
| Liver | 1.36* | 1.25 – 1.47 | 1.34* | 1.22 – 1.49 | 1.35* | 1.21 – 1.51 |
| Cervical | 1.43* | 1.34 – 1.52 | 1.37* | 1.27 – 1.48 | 1.32* | 1.21 – 1.44 |
| Prostate | 1.25* | 1.17 – 1.34 | 1.25* | 1.15 – 1.36 | 1.26* | 1.15 – 1.38 |
| Leukemia | 1.55* | 1.45 – 1.65 | 1.62* | 1.51 – 1.74 | 1.65* | 1.53 – 1.79 |
| Stomach | 1.28* | 1.19 – 1.38 | 1.27* | 1.14 – 1.40 | 1.26* | 1.12 – 1.42 |
| Ovarian | 1.10* | 1.01 – 1.20 | 1.12* | 1.02 – 1.23 | 1.14* | 1.03 – 1.26 |

**p*-value<0.05

**Supplementary Table 2. Univariate analysis on the predictors of treatment delay of more than 90 days among patients with common cancers, stratified by year at diagnosis**

| Variables | 1990 – 1999 | | | 2000 – 2012 | | |
| --- | --- | --- | --- | --- | --- | --- |
|  | *Delay* (%)  *n*=4,156  (21.5%) | RR | 95% CI | *Delay* (%)  *n*=16,498  (19.9%) | RR | 95% CI |
| *Age at diagnosis* |  |  |  |  |  |  |
| 0-19 years | 35.8 | 1 |  | 35.3 | 1 |  |
| 20-39 years | 21.9 | 0.61* | 0.46 – 0.79 | 18.2 | 0.51* | 0.42 – 0.62 |
| 40-64 years | 21.1 | 0.58* | 0.46 – 0.75 | 20.1 | 0.56* | 0.47 – 0.67 |
| 65+ years | 20.1 | 0.56* | 0.42 – 0.73 | 19.7 | 0.55* | 0.46 – 0.67 |
| *Sex* |  |  |  |  |  |  |
| Female | 20.4 | 1 |  | 18.7 | 1 |  |
| Male | 26.3 | 1.28* | 1.12 – 1.47 | 25.4 | 1.36* | 1.26 – 1.46 |
| *Residence location* |  |  |  |  |  |  |
| City | 22.2 | 1 |  | 19.9 | 1 |  |
| Municipality | 18.1 | 0.81* | 0.68 – 0.97 | 20.0 | 1.00 | 0.92 – 1.09 |
| *Hospital types* |  |  |  |  |  |  |
| Private | 19.9 | 1 |  | 17.0 | 1 |  |
| Not Hospitalized | - | - | - | 25.8 | 1.51* | 1.14 – 2.01 |
| Specialty | 19.1 | 0.96 | 0.63 – 1.45 | 22.3 | 1.30* | 1.11 – 1.54 |
| Non-Governmental Organization | 25.1 | 1.25* | 1.09 – 1.44 | 25.3 | 1.48* | 1.37 – 1.61 |
| Local Government | 15.0 | 0.75 | 0.46 – 1.20 | 17.3 | 1.01 | 0.87 – 1.19 |
| National Government | 24.1 | 1.21* | 1.04 – 1.41 | 25.1 | 1.47* | 1.36 – 1.59 |
| *Cancer stages* |  |  |  |  |  |  |
| Stage I | 18.1 | 1 |  | 11.3 | 1 |  |
| Stage II | 21.4 | 1.18 | 0.93 – 1.49 | 16.8 | 1.48* | 1.29 – 1.69 |
| Stage III | 18.6 | 1.02 | 0.85 – 1.23 | 16.1 | 1.41* | 1.25 – 1.58 |
| Stage IV | 27.6 | 1.52* | 1.28 – 1.80 | 30.1 | 2.64* | 2.37 – 2.94 |
| *Initial treatment* |  |  |  |  |  |  |
| Surgery | 14.9 | 1 |  | 12.0 | 1 |  |
| Radiotherapy | 34.1 | 2.28* | 2.01 – 2.59 | 36.5 | 3.03* | 2.83 – 3.24 |
| Chemotherapy | 40.5 | 2.71* | 2.32 – 3.16 | 37.6 | 3.12* | 2.88 – 3.38 |
| *Cancer types* |  |  |  |  |  |  |
| Thyroid | 16.7 | 1 |  | 11.0 | 1 |  |
| Breast | 21.3 | 1.27* | 1.00 – 1.61 | 18.9 | 1.71* | 1.49 – 1.96 |
| Lung | 29.1 | 1.74* | 1.32 – 2.30 | 32.5 | 2.94* | 2.53 – 3.41 |
| Colorectal | 16.6 | 0.99 | 0.59 – 1.66 | 13.3 | 1.21 | 0.92 – 1.58 |
| Liver | 15.2 | 0.91 | 0.44 – 1.86 | 32.8 | 2.97* | 2.28 – 3.86 |
| Cervical | 24.7 | 1.48* | 1.13 – 1.93 | 25.5 | 2.30* | 1.99 – 2.67 |
| Prostate | 24.1 | 1.44* | 1.05 – 1.96 | 22.4 | 2.03* | 1.71 – 2.40 |
| Leukemia | 41.5 | 2.48* | 1.85 – 3.32 | 43.8 | 3.97* | 3.31 – 4.75 |
| Stomach | 10.0 | 0.59 | 0.31 – 1.12 | 16.0 | 1.45* | 1.11 – 1.90 |
| Ovarian | 10.2 | 0.61* | 0.40 – 0.92 | 13.5 | 1.22* | 1.01 – 1.47 |

**p*-value<0.05

**Supplementary Table 3. Univariate analysis on the predictors of treatment delay of more than 90 days among patients with common cancers, stratified by residence location**

| Variables | City | | | Municipality | | |
| --- | --- | --- | --- | --- | --- | --- |
|  | *Delay* (%)  *n*=3,542  (20.4%) | RR | 95% CI | *Delay* (%)  *n*=640  (19.6%) | RR | 95% CI |
| *Age at diagnosis* |  |  |  |  |  |  |
| 0-19 years | 36.1 | 1 |  | 32.3 | 1 |  |
| 20-39 years | 19.0 | 0.52* | 0.44 – 0.62 | 19.4 | 0.60* | 0.40 – 0.88 |
| 40-64 years | 20.4 | 0.56* | 0.48 – 0.66 | 19.7 | 0.61* | 0.42 – 0.87 |
| 65+ years | 20.0 | 0.55* | 0.47 – 0.65 | 18.0 | 0.55* | 0.37 – 0.82 |
| *Sex* |  |  |  |  |  |  |
| Female | 19.0 | 1 |  | 19.1 | 1 |  |
| Male | 26.2 | 1.38* | 1.29 – 1.47 | 21.9 | 1.14 | 0.96 – 1.36 |
| *Hospital types* |  |  |  |  |  |  |
| Private | 17.7 | 1 |  | 16.4 | 1 |  |
| Not Hospitalized | 21.9 | 1.23 | 0.86 – 1.74 | 27.9 | 1.69* | 1.03 – 2.77 |
| Specialty | 21.9 | 1.23* | 1.04 – 1.45 | 21.0 | 1.27 | 0.88 – 1.83 |
| Non-Governmental Organization | 25.3 | 1.42* | 1.32 – 1.53 | 25.2 | 1.53* | 1.25 – 1.87 |
| Local Government | 16.9 | 0.95 | 0.81 – 1.11 | 18.6 | 1.13 | 0.75 – 1.71 |
| National Government | 25.7 | 1.44* | 1.33 – 1.56 | 22.7 | 1.38* | 1.17 – 1.62 |
| *Year at diagnosis* |  |  |  |  |  |  |
| 1990 – 1999 | 22.2 | 1 |  | 18.1 | 1 |  |
| 2000 – 2012 | 19.9 | 0.89* | 0.83 – 0.96 | 20.0 | 1.10 | 0.91 – 1.32 |
| *Cancer stages* |  |  |  |  |  |  |
| Stage I | 13.2 | 1 |  | 12.1 | 1 |  |
| Stage II | 17.2 | 1.30* | 1.15 – 1.48 | 19.1 | 1.57* | 1.17 – 2.09 |
| Stage III | 16.3 | 1.23* | 1.11 – 1.37 | 17.8 | 1.46* | 1.14 – 1.88 |
| Stage IV | 30.3 | 2.29* | 2.08 – 2.53 | 25.3 | 2.08* | 1.63 – 2.66 |
| *Initial treatment* |  |  |  |  |  |  |
| Surgery | 12.7 | 1 |  | 11.8 | 1 |  |
| Radiotherapy | 35.9 | 2.81* | 2.63 – 3.00 | 36.1 | 3.05* | 2.61 – 3.57 |
| Chemotherapy | 37.8 | 2.96* | 2.74 – 3.19 | 39.4 | 3.34* | 2.81 – 3.97 |
| *Cancer types* |  |  |  |  |  |  |
| Thyroid | 12.5 | 1 |  | 9.6 | 1 |  |
| Breast | 19.3 | 1.53* | 1.35 – 1.74 | 20.1 | 2.08* | 1.51 – 2.88 |
| Lung | 33.0 | 2.63* | 2.28 – 3.03 | 25.0 | 2.59* | 1.77 – 3.79 |
| Colorectal | 13.7 | 1.09 | 0.84 – 1.42 | 14.1 | 1.46 | 0.79 – 2.68 |
| Liver | 29.5 | 2.35* | 1.81 – 3.06 | 23.0 | 2.39* | 1.11 – 5.15 |
| Cervical | 25.1 | 2.00* | 1.74 – 2.30 | 26.4 | 2.74* | 1.93 – 3.89 |
| Prostate | 23.0 | 1.83* | 1.56 – 2.15 | 21.2 | 2.20 | 1.43 – 3.39 |
| Leukemia | 44.3 | 3.53* | 3.00 – 4.16 | 36.0 | 3.37* | 2.43 – 5.74 |
| Stomach | 14.4 | 1.14 | 0.87 – 1.51 | 16.4 | 1.70 | 0.95 – 3.06 |
| Ovarian | 12.8 | 1.02 | 0.85 – 1.23 | 13.4 | 1.39 | 0.89 – 2.17 |

**p*-value<0.05

**Supplementary Table 4. Univariate analysis on the predictors of treatment delay of more than 90 days among patients with common cancers, stratified by hospital type**

| Variables | Private | | | Non-Governmental Organization | | | Local Governmental | | | National Government | | |
| --- | --- | --- | --- | --- | --- | --- | --- | --- | --- | --- | --- | --- |
|  | *Delay* (%)  *n*=2,107  (17.5%) | RR | 95*%* CI | *Delay* (%)  *n*=891  (25.3%) | RR | 95*%* CI | *Delay* (*%*) *n*=154  (17.0%) | RR | 95*%* CI | *Delay* (*%*)  *n*=862  (24.9%) | RR | 95*%* CI |
| *Age at diagnosis* |  |  |  |  |  |  |  |  |  |  |  |  |
| 0-19 years | 29.6 | 1 |  | 51.7 | 1 |  | 22.2 | 1 |  | 34.0 | 1 |  |
| 20-39 years | 15.7 | 0.53* | 0.40 – 0.69 | 23.5 | 0.45* | 0.35 – 0.58 | 12.6 | 0.56 | 0.21 – 1.48 | 25.1 | 0.73 | 0.54 – 1.00 |
| 40-64 years | 17.3 | 0.58* | 0.45 – 0.74 | 25.5 | 0.49* | 0.39 – 0.61 | 18.2 | 0.82 | 0.34 – 1.98 | 24.7 | 0.72* | 0.54 – 0.96 |
| 65+ years | 18.8 | 0.63* | 0.49 – 0.81 | 22.1 | 0.42* | 0.33 – 0.55 | 16.5 | 0.74 | 0.29 – 1.90 | 23.7 | 0.69* | 0.50 – 0.96 |
| *Sex* |  |  |  |  |  |  |  |  |  |  |  |  |
| Female | 15.8 | 1 |  | 24.3 | 1 |  | 16.0 | 1 |  | 24.7 | 1 |  |
| Male | 24.5 | 1.55* | 1.42 – 1.68 | 31.1 | 1.28* | 1.10 – 1.48 | 25.8 | 1.60* | 1.09 – 2.34 | 26.3 | 1.06 | 0.90 – 1.25 |
| *Residence location* |  |  |  |  |  |  |  |  |  |  |  |  |
| City | 17.7 | 1 |  | 25.3 | 1 |  | 16.9 | 1 |  | 25.7 | 1 |  |
| Municipality | 16.4 | 0.92 | 0.82 – 1.03 | 25.2 | 0.99 | 0.83 – 1.19 | 18.6 | 1.10 | 0.72 – 1.68 | 22.7 | 0.88 | 0.76 – 1.01 |
| *Year at diagnosis* |  |  |  |  |  |  |  |  |  |  |  |  |
| 1990 – 1999 | 19.9 | 1 |  | 25.1 | 1 |  | 15.0 | 1 |  | 24.1 | 1 |  |
| 2000 – 2012 | 17.0 | 0.85* | 0.77 – 0.93 | 25.3 | 1.01 | 0.88 – 1.15 | 17.3 | 1.15 | 0.70 – 1.88 | 25.1 | 1.04 | 0.90 – 1.20 |
| *Cancer stages* |  |  |  |  |  |  |  |  |  |  |  |  |
| Stage I | 10.6 | 1 |  | 17.1 | 1 |  | 9.8 | 1 |  | 19.8 | 1 |  |
| Stage II | 13.5 | 1.26* | 1.06 – 1.51 | 21.2 | 1.23 | 0.97 – 1.56 | 20.7 | 2.11* | 1.18 – 3.78 | 24.6 | 1.24* | 1.00 – 1.56 |
| Stage III | 13.5 | 1.26* | 1.10 – 1.46 | 21.6 | 1.26* | 1.03 – 1.54 | 16.3 | 1.66* | 1.00 – 2.79 | 19.9 | 1.00 | 0.82 – 1.23 |
| Stage IV | 27.3 | 2.55* | 2.25 – 2.89 | 36.0 | 2.10* | 1.72 – 2.56 | 29.6 | 3.01* | 1.77 – 5.10 | 31.6 | 1.59* | 1.30 – 1.95 |
| *Initial treatment* |  |  |  |  |  |  |  |  |  |  |  |  |
| Surgery | 10.6 | 1 |  | 17.3 | 1 |  | 12.3 | 1 |  | 15.3 | 1 |  |
| Radiotherapy | 32.9 | 3.09* | 2.83 – 3.37 | 39.4 | 2.27* | 2.00 – 2.57 | 41.2 | 3.33* | 2.34 – 4.73 | 39.8 | 2.60* | 2.28 – 2.95 |
| Chemotherapy | 32.8 | 3.08* | 2.78 – 3.41 | 50.5 | 2.90* | 2.52 – 3.35 | 38.0 | 3.07* | 2.15 – 4.37 | 44.9 | 2.93* | 2.51 – 3.42 |
| *Cancer types* |  |  |  |  |  |  |  |  |  |  |  |  |
| Thyroid | 10.3 | 1 |  | 15.6 | 1 |  | 4.3 | 1 |  | 16.4 | 1 |  |
| Breast | 16.1 | 1.56* | 1.32 – 1.84 | 26.1 | 1.66* | 1.30 – 2.13 | 17.4 | 4.05* | 1.68 – 9.76 | 25.7 | 1.56* | 1.22 – 1.98 |
| Lung | 31.0 | 3.00* | 2.51 – 3.60 | 34.3 | 2.19* | 1.16 – 2.97 | 33.3 | 7.73* | 2.52 – 9.64 | 28.4 | 1.72* | 1.25 – 2.37 |
| Colorectal | 13.7 | 1.32 | 0.98 – 1.79 | 17.2 | 1.10 | 0.59 – 2.02 | 10.7 | 2.48 | 0.63 – 9.79 | 14.2 | 0.86 | 0.46 – 1.60 |
| Liver | 28.5 | 2.76* | 2.02 – 3.77 | 31.5 | 2.01* | 1.00 – 4.06 | 37.5 | 8.70* | 2.51 – 10.05 | 20.0 | 1.21 | 0.49 – 2.99 |
| Cervical | 18.2 | 1.76* | 1.44 – 2.16 | 28.5 | 1.82* | 1.40 – 2.37 | 38.0 | 8.82* | 3.55 – 10.87 | 31.7 | 1.92* | 1.50 – 2.47 |
| Prostate | 23.1 | 2.24* | 1.84 – 2.73 | 26.9 | 1.72* | 1.20 – 2.45 | 28.5 | 6.62* | 2.22 – 9.76 | 22.8 | 1.38 | 0.91 – 2.10 |
| Leukemia | 36.5 | 3.54* | 2.83 – 4.42 | 57.6 | 3.67* | 2.73 – 4.94 | 29.4 | 6.82* | 2.20 – 10.14 | 49.3 | 2.99* | 2.18 – 4.10 |
| Stomach | 12.9 | 1.25 | 0.88 – 1.77 | 20.5 | 1.31 | 0.78 – 2.21 | 21.0 | 4.88* | 1.43 – 8.58 | 16.9 | 1.02 | 0.58 – 1.79 |
| Ovarian | 12.8 | 1.24 | 0.98 – 1.58 | 14.5 | 0.92 | 0.65 – 1.31 | 5.0 | 1.16 | 0.32 – 4.18 | 13.6 | 0.82 | 0.57 – 1.18 |

**p*-value<0.05
